# Acceptability of the WHO labor care guide among midwives in Tanzania: an exploratory qualitative study

**DOI:** 10.3389/fpubh.2026.1739655

**Published:** 2026-03-16

**Authors:** Nyabwire Joel Lukumai, Israel P. Nyarubeli

**Affiliations:** 1 School of Public Health and Social Sciences, Muhimbili University of Health and Allied Sciences, Dar es Salaam, Tanzania; 2 Department of Psychosocial Sciences, Faculty of Psychology, University of Bergen, Bergen, Norway

**Keywords:** acceptability, intrapartum care, labor care guide, midwives, qualitative study, Tanzania

## Abstract

**Introduction:**

The World Health Organization’s Labor Care Guide (LCG) is a next-generation partograph designed to improve intrapartum care by promoting accurate monitoring, critical thinking, and woman-centered practices. Evidence from low- and middle-income countries suggests that the LCG is generally practicable and acceptable among maternity care providers. Tanzania adopted the LCG in 2023, but little is known about its acceptability among frontline midwives in routine practice.

**Methods:**

We conducted an exploratory qualitative study in a Municipality located in northwestern Tanzanian between May and June 2025. Twenty participants (midwives) from public health facilities were purposively selected. Data were collected through in-depth interviews guided by the Theoretical Framework of Acceptability (TFA), focusing on perceived effectiveness, burden, and intervention coherence. Interviews were transcribed, translated, and analyzed using Braun and Clarke’s thematic analysis approach.

**Results:**

Three major themes emerged: (i) *perceived effectiveness,* participants viewed the LCG as a structured tool that could support labor monitoring (systematic assessment of fetal heart rate, labor progress, and early detection of complications), facilitates timely decision-making, which could support maternal and neonatal outcomes; (2) *perceived burden,* increased documentation workload and time demands compared to the traditional partograph, particularly under staffing constraints; and (3) i*ntervention coherence,* most participants understood the LCG’s objectives and alignment with clinical practice, though challenges with some unfamiliar notation and contraction assessment were noted.

**Conclusion:**

Participants generally perceived the LCG as acceptable tool for improving intrapartum care. However, contextual barriers such as increased workload, inadequate staffing, and integration into existing workflows were highlighted. Policy efforts should prioritize on-the-job training, workflow adaptation, and regular supportive supervision. Future research should employ mixed-methods and longitudinal designs to assess sustained LCG acceptability and effect on maternal and neonatal outcomes.

## 1 Introduction

Complications during labor and the first 24-h postpartum contribute substantially to the global burden of preventable maternal and neonatal deaths, particularly in low- and middle-income countries (1). In 2023, approximately 260,000 women died due to complications related to pregnancy and childbirth (equivalent to one death every 2 min). Additionally, 1.9 million babies were stillborn, and 2.3 million newborns died within their first month of life, accounting for nearly 47% of all under-five deaths globally (2). Improving the quality, safety, and person-centeredness of intrapartum care remains pivotal (3). In 2020, World Health Organization (WHO) published the Labor Care Guide (LCG), a tool to replace the traditional partograph, intending to provide structured support for healthcare providers during labor and childbirth (4, 5). The LCG was designed to improve decision-making, enhance maternal and fetal monitoring, and ultimately improve outcomes by standardizing labor management practices (6, 7). One of the primary objectives for implementing the LCG is the improvement in maternal and neonatal health outcomes. It enables healthcare providers to monitor labor progress systematically and identify deviations from the normal trajectory. This early detection of complications, such as prolonged labor, postpartum hemorrhage, or fetal distress, facilitates timely interventions, reducing maternal morbidity and mortality (4). Since its introduction, there has been growing interest by various countries’ healthcare institutions to implement, adopt this tool to improve women’s overall experience and quality of care during labor and delivery. According to WHO, the LCG enhances evidence-based, woman-centered care. Its manual provides guidance on intended use, initiation, frequency, and documentation standards and adaptation for labor wards (8).

Evidence from research with maternity care providers indicates that the LCG is generally feasible and acceptable and can support more precise monitoring and critical thinking rather than encouraging rote documentation or a “tick-box” approach (9, 10). For instance, maternity care providers described the LCG as supporting precise monitoring, encouraging critical thinking, and enhancing provision of woman-centered care, with a reported median usability score of 67.5% (9). Some design improvements, such as increasing the cell size for documentation, could further enhance the usability of the LCG (9). A study that was conducted across Asia, Europe, Africa and America revealed that contents in the LCG were usually seen as clear, relevant, and recorded at an adequate frequency, with most sections deemed complete (11).

Tanzania has made a remarkable gain in maternal health in recent years, with routine and survey data indicating substantial declines in the maternal mortality ratio (MMR) and high policy commitment to strengthening intrapartum services, though absolute burdens remain non-trivial, and measurement differences persist across sources (12, 13). Neonatal outcomes have also improved; however neonatal deaths remain a critical share of under-five mortality and improving the quality and timeliness of intrapartum care is necessary to accelerate progress (13). In 2023, Tanzania adopted and developed its own LCG manual for skilled health care providers who care for women during labor and childbirth. Nevertheless, evidence on how frontline midwives experience and accept the routine use of LCG in lower-level health facilities remains limited. In public primary care maternity settings, midwives frequently manage high caseloads with limited staffing and inconsistent supportive supervision. Under these conditions, LCG acceptability becomes consequential, as perceived effectiveness may drive its adoption as a decision support tool, while perceived burden especially documentation time and workflow disruption may shape fidelity, completeness, and sustained routine use. In exploring the acceptability of the LCG among midwives in Tanzania, we used Sekhon’ Theoretical Framework of Acceptability (TFA) (14) as an interpretive lens, focusing on perceived effectiveness, burden, and intervention coherence, the three constructs that were most salient to midwives’ accounts and that map closely to implementation success at lower health facilities level. Accordingly, this study sought to examine “*how do frontline midwives in public primary-care maternity settings in Tanzania perceive the acceptability of the WHO - LCG in routine intrapartum care, specifically in terms of perceived effectiveness, perceived burden, and intervention coherence and what contextual and workforce conditions shape these perceptions*?”

### 1.1 Theoretical framework of acceptability

To conceptualize acceptability, this study draws on a Sekhon et al.’s TFA, applied with analytic focus on selected constructs (15), which defines acceptability as a multi-faceted construct reflecting the extent to which midwives delivering an intervention (e.g., the LCG implementation) consider it appropriate, based on anticipated or experienced cognitive and emotional responses. For this study, *acceptability was referred to the extent to which midwives consider the WHO – LCG an appropriate and feasible tool for routine intrapartum care, based on their cognitive understanding and professional responses to its use. It encompasses perceptions of whether the LCG aligns with professional values, is practical within existing workflows, and is likely to achieve its intended purpose of improving maternal and neonatal outcomes*. The TFA primarily includes seven apparent constructs such as affective attitude, burden, ethicality, intervention coherence, opportunity costs, perceived effectiveness, and self-efficacy. For this study, three domains were most salient for acceptability. These constructs are: Perceived effectiveness (the extent to which midwives believe the LCG achieves its intended purpose of improving labor monitoring and outcomes), burden (the perceived effort required to implement the LCG within routine workflows), and intervention coherence (the degree to which midwives understand the LCG, and how it fits with existing practices and values). This theoretical insight strengthens interpretation of qualitative findings beyond descriptive accounts by organizing midwives accounts into theoretically meaningful dimensions. In this respect, we used TFA to guide both data collection and analysis. The interview guide incorporated prompts aligned with three constructs most relevant to LCG implementation such as perceived effectiveness, burden, and intervention coherence while remaining open to other emergent dimensions (Table 1).

**Table 1:** Operationalization of theoretical framework of acceptability (TFA) for the WHO- labor care guide (LCG).

TFA construct	Definition	Operationalized meaning in the study	Example of interview questions
Perceived effectiveness	The extent to which the intervention is perceived as likely to achieve its purpose	Midwives’ beliefs that LCG supports timely detection of complications, improves decision-making, and enhances quality of intrapartum care	In your experience, how has using the LCG affected the way you monitor labor and make decisions? Probes- *Can you describe a situation where the LCG helped you notice a problem earlier?* *Which sections of the LCG are most helpful for decision-making?* *Has the LCG changed how you respond to slow progress or fetal distress?*
Burden	Perceived amount of effort that is required to participate in the intervention	The effort, time and workload needed to document observations in LCG and perform required assessments, particularly under staffing constraints and during multiple concurrent labor, and competing clinical tasks and system workflows.	What effort or extra work, if any, does the LCG require compared with the partograph? Probes: - *Which sections take the most time to complete?* *How do staffing levels or multiple laboring women affect your ability to complete the LCG?* *What do you do when you must prioritize urgent care over documentation?”*
Intervention coherence	The extent to which the participants understand the intervention and how it works	Midwives understand the LCG’s purpose, structure, and intended use of the LCG, and how well it fits with routine clinical workflows, including clarity of symbols, procedures and expectation for documentation.	What do you understand to be the main purpose of the LCG, and how is it supposed to work in practice? *Probes: -Which parts are clear or unclear?* *Are there symbols or steps that are confusing?* *How did training prepare you to use the LCG?* *What support would improve your understanding or confidence?”*

## 2 Materials and methods

### 2.1 Study design

We conducted an exploratory qualitative study using embedded single-case design, to examine midwives’ perceptions on the acceptability of the WHO - LCG during routine intrapartum care within healthcare facilities. The “case” was defined as a bounded municipal implementation setting in which the WHO - LCG had been introduced following its national rollout and was in active routine use for intrapartum care within public primary healthcare services during the study period. The primary unit of analysis was frontline midwives’ experiences and perceptions of using the LCG, while public primary healthcare facilities within the municipality (dispensaries and health centers providing intrapartum care) constituted embedded units that shaped implementation conditions such as workload, staffing, and workflow. A case study approach was selected because the phenomenon under investigation WHO-LCG acceptability in routine intrapartum care, is context-dependent and occurs in real-world clinical environments where boundaries between the intervention (WHO-LCG acceptability), the actors (frontline midwives), and the implementation context (primary healthcare facility providing intrapartum care) are intertwined. In addition, empirical evidence on LCG implementation experiences in similar lower-level public facilities following national rollout remains limited, warranting in depth exploration to generate contextually grounded insights (16). This methodological choice aligns with the study’s objective of exploring the acceptability of LCG through rich, detailed accounts obtained via in-depth interviews, allowing for refined understandings into midwives’ practical experiences and interpretations (16). Furthermore, we used TFA as a sensitizing lens rather than a prescriptive coding structure. The case study design enabled in-depth examination of acceptability as a context dependent implementation phenomenon in real-life routine primary-care maternity settings, while the TFA constructs helped structure interpretation of midwives’ accounts around theoretically meaningful dimensions of acceptability. This approach allowed us to generate themes inductively from the data and then interpret them in relation to perceived effectiveness, burden, and intervention coherence, as the constructs that consistently characterized participants’ accounts and best illuminated the study aims. To minimize conceptual ambiguity, we adopted an application of selected TFA constructs, focusing exclusively on three domains in this study. The study is reported in accordance with the Consolidated criteria for reporting qualitative studies (COREQ) checklist (17) (Supplementary File S1).

### 2.2 Study context

The study was conducted in public health facilities within a municipality located along the southern shores of Lake Victoria in northwestern Tanzania. During the study period, the municipality comprised 68 health facilities, of which 46 provided reproductive, maternal, newborn and child health services. Tanzania adopted the WHO -LCG in 2023, and during the study period the WHO-LCG was the recommended labor monitoring tool in public facilities following the national rollout and related trainings and orientations.

The municipality was purposively selected as an information-rich case based on three criteria. First, it represented early routine adoption of the WHO-LCG following national rollout, ensuring that midwives participated in practical experience using the tool in routine intrapartum care. Second, the municipality includes a mix of both rural and urban healthcare service delivery contexts, enabling exploration of how acceptability is shaped across heterogeneous environment, workload and resource conditions within the same administrative health system. Third, administrative coherence within a single municipal health system provided a consistent governance and reporting environment, strengthening the interpretability of facility-level differences as embedded contextual variation rather than cross-district variation. These criteria directly informed our purposive sampling of midwives with direct LCG exposure and the use of in-depth interviews to elicit detailed accounts of perceived effectiveness, perceived burden, and intervention coherence in routine practice. Conceptualizing the municipality as a case provided analytic leverage to examine how municipal rollout conditions and primary care organizational realities shaped acceptability, while embedded facility units enabled within case comparison of how workload, staffing, and workflow differences influenced perceived effectiveness, burden, and coherence.

### 2.3 Study participants and selection

The study participants comprised midwives working in maternity wards of public health facilities. These midwives were actively engaged in providing intrapartum care, making them suitable informants for exploring the acceptability of the LCG in routine clinical practice. All selected facilities, i.e., health centers and dispensaries, offer maternity services and represent primary points of care for labor and delivery in the municipality. These facilities serve a heterogeneous population of women during labor and delivery and hence reflect systemic and practical experiences in implementing new clinical tools such as the LCG (18).

A purposive sampling strategy was employed to recruit a minimum of 20 midwives for this study with balanced distribution across high and low volume health facilities (i.e., 10 – participants each) with a mix between rural and urban (19, 20). This sampling strategy (rural–urban; high-low volume health facility deliveries) was selected to address the study question by capturing variation in implementation conditions likely to shape LCG acceptability. Participants were selected based on their direct involvement in intrapartum care and prior exposure to the LCG training provided by the Ministry of Health. To ensure informed perspectives, only midwives with at least 1 year of clinical experience in maternity wards were included. A sample size of 20 was deemed sufficient for an exploratory qualitative case study to capture diverse viewpoints while remaining manageable for in-depth analysis (16). Importantly, data saturation, i.e., the point at which no new themes emerged, served as the guiding principle for determining adequacy (21). Saturation was assessed across the full dataset before thematic consolidation and interpretive organization into the three TFA-aligned themes. This approach aligns with qualitative research standards that prioritize richness and depth of information over statistical representativeness.

### 2.4 Data collection

Between early May and June 2025, 20 in-depth interviews (IDIs) were conducted with midwives working in maternity wards across 10 purposively selected healthcare facilities. Health facilities were selected to capture a rural–urban mix and variation in health facility delivery volume. Ten participants were recruited from high-volume and ten from low-volume facilities. All participants provided informed written consent for audio recording, and no dropouts were experienced during the entire period of interviews. The interviews were guided by a semi-structured interview guide developed in English and translated into Swahili and piloted to ensure clarity and cultural relevance. The participants were midwives of varying educational backgrounds and professional experience, ranging from certificate holders to bachelor’s degree graduates, with years of service spanning from 1 to 27 years (Table 2). Each interview lasted approximately 45–60 min and was conducted in a private setting within the health facility to ensure confidentiality and comfort. The interviews were moderated by the researcher (NJL), trained in qualitative interviews, who, in addition, documented field notes to capture non-verbal cues and contextual observations. All interviews were audio-recorded to ensure accurate transcription and analysis.

**Table 2:** Description of participant information (midwives) involved in in depth interview in Tanzania.

**IDI no.**	**Participant age (yrs)**	**Education level**	**Work experience (yrs)**
1	30	Diploma	1
2	28	Diploma	2
3	31	Diploma	10
4	48	Diploma	23
5	37	Diploma	2
6	43	Certificate	2
7	49	Certificate	7
8	35	Diploma	10
9	35	Diploma	12
10	47	Diploma	17
11	28	Degree	1
12	34	Diploma	13
13	29	Diploma	1
14	53	Diploma	20
15	38	Diploma	13
16	51	Degree	27
17	48	Degree	23
18	44	Degree	19
19	39	Diploma	13
20	45	Diploma	20

Given the researcher’s academic background in public health and maternal care, and with previous nursing experience, careful attention was paid to positionality and the potential influence on the data collection process. During data collection she was not a practicing midwife and held no managerial or supervisory role over participants. This positioning enabled shared professional language while also carrying a risk of preconceptions about the LCG given its endorsement in global maternal health policy. To minimize positional bias and social desirability effects, we adopted a reflexive approach throughout the study (22). The interviewer used neutral, open-ended questions and non-leading probes, explicitly emphasized that there were no “right” or “wrong” answers, and prioritized participants own voices of LCG implementation experiences and acceptability during analysis (23). Interviews were conducted in private, conducive settings within the facilities, with assurances of confidentiality and no linkage to supervisors. Reflexive bracketing was supported through a reflexive journal maintained throughout data collection and analysis to document assumptions and analytic decisions.

Thematic saturation was determined iteratively during the analysis phase. After each interview, preliminary coding and theme identification were conducted. By the twentieth interview, no new themes or information were emerging, indicating that data saturation had been reached. Participants did not review transcripts or provide feedback on the findings due to time constraints and workload; however, data credibility was ensured through iterative team discussions and validation of emerging themes (24). This was confirmed through team discussions and cross-validation of patterns, ensuring that the data adequately addressed the study objectives related to the acceptability of the LCG among midwives.

### 2.5 Data analysis

We analyzed interview transcripts using thematic analysis following Braun and Clarke’s six-phase approach, with the Theoretical Framework of Acceptability (TFA) used as an analytic lens to sensitize interpretation to three focal domains, namely perceived effectiveness, perceived burden, and intervention coherence (25). All interviews were audio-recorded, transcribed verbatim, and transcripts were read repeatedly to support familiarization, noting initial impressions, recurring phrases, and variations. Initial open coding was conducted manually and systematically across the dataset, assigning concise labels to meaning units capturing experiences of LCG use. First, transcripts were read repeatedly to support familiarization. Second, we conducted manual, line-by-line coding, assigning concise labels to meaning units that captured key aspects of LCG use (e.g., “*structured monitoring prompts*,” “*time pressure after vaginal examination*,” “*confusion with LCG symbols*,” and “*continuity across shifts*”). Third, related codes were compared and clustered into broader categories reflecting patterned meanings across participants (e.g., “*decision support*,” “*documentation workload*,” and “*procedural clarity*”). Fourth, we generated candidate themes by examining relationships across categories and organizing them in relation to the TFA lens, resulting in three overarching themes. Fifth, themes were iteratively reviewed against the full dataset to ensure internal coherence and clear distinction between themes, with supporting quotations checked for appropriateness and representativeness. Finally, themes were refined, named, and defined with explicit boundaries, and are presented with illustrative quotations to maintain a transparent link between participants’ accounts and the analytic interpretation. Codes were iteratively compared and collated into broader categories, and a preliminary visual coding tree was developed to organize related codes under candidate thematic areas aligned with the TFA domains (26).

Theme development proceeded recursively, moving between coded extracts, categories, and the full dataset to ensure that themes were internally coherent, clearly distinct, and strongly supported by the data. To enhance analytical rigor, themes were reviewed and refined through a two-stage process, first, NJL checked candidate themes against the raw extracts to ensure conceptual fit and boundary clarity; second, NJL and IPN discussed the evolving codebook, coding decisions, and theme structure to challenge interpretations and resolve discrepancies through reference back to the transcripts. Information saturation (thematic) was assessed prospectively and iteratively alongside analysis using a saturation tracking log that documented the emergence of new codes after each interview (21). From approximately 18 interviews onwards, no substantively new codes or dimensions relevant to the three TFA domains were identified. Subsequent interviews primarily elaborated and confirmed existing codes and categories rather than extending the thematic structure. Between interviews 18 and 20, accounts repeatedly reflected the same patterns regarding perceived effectiveness for labor monitoring and decision-making, documentation workload and staffing-related challenges shaping perceived burden and understanding of LCG objectives alongside challenges with specific components such as contraction assessment and unfamiliar notation. The final interview primarily elaborated and confirmed existing categories rather than extending the thematic structure. Recruitment therefore ceased at 20 interviews when additional data were judged likely to yield redundancy rather than new analytical insights. The themes were systematically organized and supported by direct quotations from participants to ensure that the analysis remained grounded in their lived experiences and accurately reflected the study objectives. Additionally, we conducted a within-case comparative analysis to examine whether coded experiences varied by education level, years of professional experience, and facility context (high- and low volume health facilities; rural and urban), thereby identifying patterned differences across participants and settings though this study was not designed for formal subgroup analyses.

To maintain trustworthiness and culturally linguistic fidelity, coding and theme development were conducted in Swahili, i.e., the interview language, and translations into English were completed only after themes were finalized for the purpose of reporting illustrative quotations in the manuscript, following best practices (27, 28). The final write-up presented the findings in a structured and coherent narrative, organized by main themes and sub-themes. Each theme was supported by direct quotations from participants to illustrate key points and ensure that the analysis remained grounded in the voices of midwives.

### 2.6 Ethical consideration

Ethical clearance for this study was obtained from the Institutional Research and Publications Committee of Muhimbili University of Health and Allied Sciences (MUHAS), reference number MUHAS-REC-04-2025-2808, dated 14 April 2025. Formal permission to conduct the research was also granted by the Municipal Council where the study was conducted. Prior to data collection, all participants were provided with a detailed explanation of the study purpose, procedures, and their rights, after which informed consent was obtained through signed consent forms. Throughout the study, strict confidentiality was maintained by anonymizing all participant data, securely storing transcripts and related materials, and ensuring that no personal identifiable information was recorded. Access to the data was restricted to the researcher, and all information collected was used solely for academic and research purposes. Participation was entirely voluntary, with participants informed of their right to withdraw at any time without consequence.

## 3 Findings

### 3.1 Theme 1: Perceived effectiveness of LCG acceptability

The participants in this study consistently perceived LCG as a valuable and effective tool for improving maternal and neonatal outcomes during childbirth. Midwives often pointed out that the LCG plays an important role in guiding decision-making processes, step by step, particularly during the second stage of labor, a period they identified as high-risk for expectant mothers and newborns. They further explained that complications such as fluid aspiration and birth asphyxia often occur when labor is prolonged or poorly monitored. In such cases, the structured guide provided in the LCG was seen as effective in detecting early warning signs and prompting timely interventions.

#### 3.1.1 Sub-theme 1.1: Usefulness of LCG in labor monitoring

Most participants consistently viewed the LCG as a valuable and practical tool for structured monitoring throughout labor. Its design was perceived to support continuous assessment of both maternal and fetal well-being, particularly during the second stage of labor, which participants identified as a critical period for detecting complications. The LCG was understood for its ability to guide midwives to systematically monitor and record key clinical indicators such as fetal heart rate, maternal blood pressure, uterine contractions, and cervical dilation. This structured approach was seen as enhancing clinical alertness and reducing the likelihood of adverse outcomes such as birth asphyxia, fluid aspiration, or delayed interventions. Participants mentioned that the LCG helped them remain alert and responsive to changes in labor progress especially in low resource settings and primary healthcare (dispensaries and some health centers) where advanced diagnostic tools and trained professionals such as ultrasound are unavailable, making the LCG a practical alternative for guiding labor assessment as noted from one participant “…… *If I measure and find 111bpm or 110 bpm, LCG will indicate that fetal distress might be occurring. This alerts me that I remain vigilant, document correctly, and intervene when necessary…”* (IDI_10, 47 years, diploma holder, 17 -years work experience). Furthermore, they noted that LCG provided a clear and useful procedure for identifying variations from expected patterns, prompting timely clinical decisions. The guide was also viewed as a supportive tool for less experienced midwives, offering a standardized format that reduced uncertainty and improved confidence in labor management. For example, midwife IDI_11 explained that,

… *“I monitor fetal heart rate and labor progress while also evaluating amniotic fluid and fetal positioning. These indicators help detect fetal distress early, preventing possible neonatal death”* (IDI_11, 28 years, degree holder, 1 -year work experience).

#### 3.1.2 Sub-theme 1.2: LCG provided structured tracking flow that supports timely decision making

Most participants consistently described the LCG as a practical guide for tracking labor progress in a systematic manner. On admission, midwives reported assessing multiple indicators such as cervical dilation, abdominal examination notes, antenatal records, and relevant clinical history which often may initially appear normal but could change as labor advances as described by one midwife participant IDI_15. Additionally, the LCG was perceived to provide a clear and structured framework for ongoing labor monitoring. For instance, vaginal examinations were done at four-hour intervals and ensuring that observations were recorded in an organized sequence.

*… “upon admission, I evaluate various indicators that may be positive or negative. For instance, a mother may arrive with normal cervical dilation, a stable abdominal examination, good antenatal records, and a healthy biological history. However, as labor progresses, certain symptoms may change. With LCG guide, we conduct vaginal examinations every four hours, recording observations systematically. If another healthcare provider takes over, they can review documented findings, analyze trends, and determine if labor is progressing well or if complications are arising”* (IDI_15, 38 years, diploma holder, 13 -year work experience).

From the participants’ perspective, this structured documentation served more than a record-keeping function; it created a shared reference point that enabled other healthcare providers to review previous notes on pregnant women’s files, identify and follow emerging trends, and make informed decisions about whether labor progressed as expected or if complications were developing. Midwives emphasized that this continuity of information across shifts improved team communication and supported timely interventions when deviations from normal patterns were detected.

However, some participants perceived LCG tool as complicated at it has many parts to fill in compared to traditional partograph due to its multiple sections and unfamiliar notation. They explained that certain components particularly those related to contraction assessment and fetal head descent were challenging to interpret. Unlike the traditional partograph, which used simple *plus* and *minus* signs, the LCG employs mathematical symbols such as “*greater than*” and “*less than*,” which some midwives found confusing during documentation. This unfamiliarity occasionally led to hesitation or was perceived as a source of errors when recording observations. For example, midwife_IDI_14 stated that,

*“It seems very complicated in some sections, for example, contraction and descent of the head, especially when you compare it to partograph. The LCG uses greater than and less than symbols, which confuses many staff members because they don’t always understand whether something is considered greater or smaller. This creates confusion, especially in tracking descent, whereas the old partograph did not have these elements. The previous tool simply used plus and minus signs”* (IDI_14, 53 years, diploma holder, 20 -years work experience).

In other circumstances, our participants acknowledged that they do not always adhere to the LCG steps in strict sequence, explaining that clinical realities often require immediate action. For instance, some women arrive at the facility fully dilated and ready to push, leaving little time to complete all recommended procedures before delivery. In such cases, midwives prioritize urgent care over documentation, as quoted below.

*“….. No, I do not always follow all steps sequentially. It depends on the condition of the mother upon arrival. Some mothers come in fully dilated and ready to push, making it difficult to strictly follow LCG guidelines since the mother is already in the pushing stage and cannot go through the recommended procedures as per LCG.”* (IDI_13, 29 years, diploma holder, 1 -year work experience).

### 3.2 Theme 2: Perceived burden for LCG acceptability

Overall, participants perceived that the LCG is a comprehensive tool designed to improve labor monitoring; however, its implementation was associated with varying levels of perceived burden. While some midwives found the components practicable, others highlighted challenges related to time, workload, and staffing constraints. The burden was most felt in facilities with few staff on duty, where midwives had to divide their attention between clinical care and detailed record-keeping.

#### 3.2.1 Sub-theme 2.1: Expanded documentation

Participants perceived that the LCG was straightforward and did not present major difficulties during routine use. Nevertheless, most participants viewed documentation effort as contributed to perceived burden shaped by staffing and workflow constraints particularly time used for documentation tasks after vaginal examinations. They emphasized that accurate and timely completion is essential for safe delivery, yet often difficult in busy maternity units. Staff shortages were repeatedly cited as a key barrier, forcing midwives to attend multiple laboring women simultaneously, which sometimes led to rushed or incomplete entries as quoted below.

*“The time required to fill out LCG is quite long. There are moments when documentation must be done immediately to avoid forgetting details, but at the same time, a mother arrives needing assistance. When I ask her to wait a little while I complete documentation, she may feel neglected, even though the reason is that LCG requires careful attention and thorough documentation of every measurement taken.”* (IDI_09, 35 years, diploma holder, 12 -years work experience).

#### 3.2.2 Sub-theme 2.2: Increased workload compared to partograph

Participants frequently compared the LCG to the traditional partograph, noting that the LCG covers a broader range of parameters from admission to discharge including additional information and elements to fill in, such as personal details, physical and abdominal examinations, vaginal assessments, postnatal care, newborn checks, and counselling. While this expansion was seen as improving completeness, it also contributed to increased workload as quoted below.

*…… “Previously, partograph charts contained fewer elements. We only monitored labor progress until delivery, documenting basic maternal information and contractions. Now, LCG is used from admission through discharge, including personal particulars, physical examinations, abdominal examinations, vaginal examinations, postnatal care, newborn examinations, companionship, decision-making and post-counseling.”* (IDI_12, 34 years, diploma holder, 13 -years work experience).

Additionally, few participants noted that unwillingness to learn or limited prior exposure often resulted in skipped sections or incorrect LCG entries as stated by IDI_04. *“…Sometimes I feel I’m too old for the new staff, compared with using patogram, the ones I’ve always worked with and feel comfortable with… (IDI_04, 48 years, diploma, 23 – years of experience).”* Participants also noted that caregivers sometimes failed to review existing medical records thoroughly, leaving out critical information such as blood group or hemoglobin levels even when documented in the mother’s card. For example, IDI_12 explained that

*“If you have more than one pregnant mother in labor and in a case, where there are few staff to support, it obvious you will monitor the mothers simultaneously by looking on who is almost 10 centimeters fully dilated and who is still 6-centimeter maybe. Therefore, this time you are too occupied and sometimes forget to check information of arriving mother on her card, for example, Hb level and blood group that are very important ….”* (IDI_02, 28 years, diploma holder, 2 – years of experience). These omissions were attributed to negligence or the pressure to complete tasks quickly during busy shifts. On the other hand, a few participants felt that the LCG streamlined their work by combining all necessary information into one form. For example, one participant said “…*No, I think LCG has made things easier…”* (IDI_11, 28 years, degree holder, 1 -year work experience).

### 3.3 Theme 3: Intervention coherence

Participants generally perceived that the LCG aligns well with existing maternal care practices and workflows. They described the tool as consistent with routine protocols and supportive of integrated service delivery. However, they noted that practical realities such as staff shortages and high caseloads sometimes affected the ability to implement the guide fully.

#### 3.3.1 Sub-theme 3.1: Understanding and alignment with intended objectives

Most participants demonstrated a clear understanding of the LCG’s purpose and objectives. Participants believed the LCG’s structured monitoring may help prevent maternal and neonatal mortality by guiding labor monitoring from the first to the fourth stage and supporting immediate postpartum care. Participants noted that the guide promotes comprehensive management, including postnatal education, breastfeeding, and newborn care.

*“The goal of LCG is to monitor labor progression from the first to the fourth stage. After that, it guides the management of the newborn and the mother, including postnatal education, breastfeeding, and overall care. Its primary aim is to reduce maternal and neonatal mortality”.* (IDI_07, 49 years, Certificate holder, 7 -years work experience).

While many participants articulated the overall purpose of the LCG, several views suggested that procedural coherence was incomplete, particularly regarding contraction assessment within 10-min intervals and interpretation of unfamiliar notation. Others noted that these difficulties were mitigated by training and familiarity with the tool. A few participants stressed that staffing shortages, rather than the tool itself, were the burden to consistent implementation.

*“For me, one of the more confusing sections is monitoring labor progress, specifically assessing contractions within 10-minute intervals and measuring duration. The process of counting and verifying contractions is somewhat complex.”* (IDI_03, 31 years, diploma holder, 10 -years work experience).

### 3.4 Linking themes to variation in education, seniority, and facility context

Across themes, participants’ accounts suggested that experiences of the LCG varied by educational background, professional seniority, and work conditions. Although the study was not designed for formal subgroup comparisons, midwives with degree levels more often described the LCG as intuitive and supportive of systematic monitoring and decision-making. For example, one participant emphasized that the LCG enabled early detection of fetal distress through structured monitoring of key indicators.

“…I monitor fetal heart rate and labor progress… These indicators help detect fetal distress early…” (IDI_11, 28 years, degree, 1-year experience).

In contrast, participants with lower education, particularly those with longer time in practice, more frequently described difficulty with unfamiliar notation and specific procedural elements such as contraction assessment and head descent tracking. As one participant noted, *“The LCG uses greater than and less than symbols… This creates confusion, especially in tracking descent…” (IDI_14, 53 years, diploma, 20-years’ experience).*

Professional seniority also appeared to shape the WHO-LCG acceptability perspectives among midwives. Less experienced midwives sometimes framed the guide as a structured support that improved confidence and continuity of documentation, particularly where clinical decision-making had to be shared across shifts. For instance, one participant explained how the structured format helped subsequent providers interpret trends and identify emerging complications: -

“…If another healthcare provider takes over, they can review documented findings, analyze trends, and determine if labor is progressing well…” (IDI_15, 38 years, diploma, 13-years’ experience).

Conversely, some midwives who have been practicing longer compared the WHO-LCG to the long-used partograph and described greater disruption related to unfamiliar symbols and expanded documentation requirements.

Overall, midwives with more recent or higher-level training more often described the LCG as aligned with systematic documentation and clinical reasoning, whereas those with longer established routines more often reported disruption due to unfamiliar notation and the expanded documentation scope. These patterns may reflect differences in training exposure, familiarity with structured recording systems, and professional routines shaped by long-standing partograph use. However, this should be interpreted as within-case patterns rather than formal subgroup effects.

**Figure 1:**
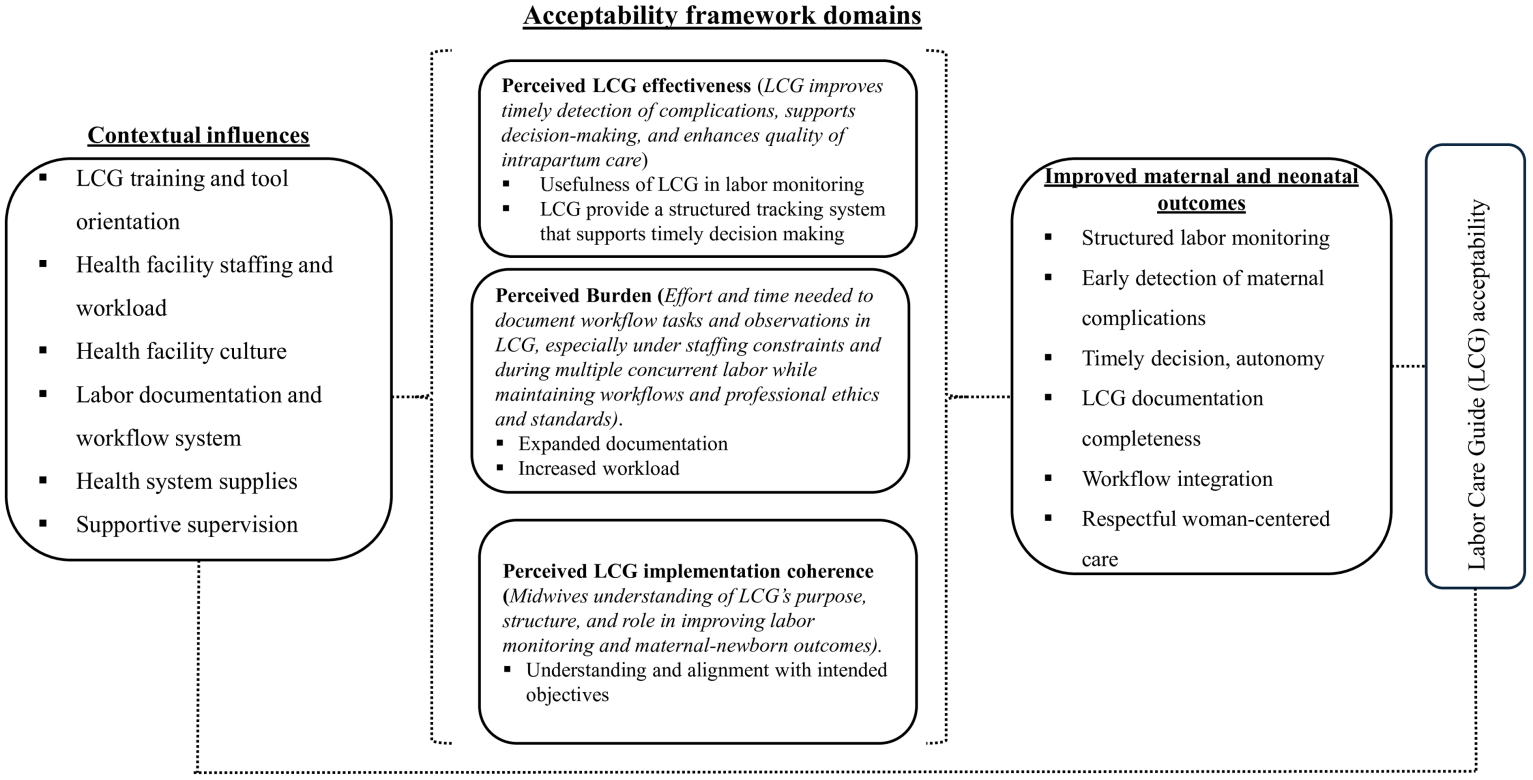
Theoretical framework of labor care guide (LCG) acceptability among midwives in Tanzania.

Generally, our findings, summarized in the theoretical framework of LCG acceptability (Figure 1), show how contextual factors and three core TFA domains (perceived effectiveness, perceived burden, and intervention coherence), shaped LCG adoption among midwives. From participants’ view, contextual inputs such as LCG tool orientation and training, adequate staffing levels, appropriate workload, proper documentation systems, adequate medicines and supplies, and supervisory support underpin successful acceptability of LCG. Participants strongly affirmed perceived effectiveness, describing the LCG as a structured tool that could support timely detection of complications, support decision-making, and enhance intrapartum care quality. In contrast, perceived burden reflected the effort and time required for documentation, particularly under staffing constraints and during multiple concurrent labors. Participants noted that while the LCG may improve documentation comprehensiveness, its length and level of detail may increase workload in resource-limited settings. Intervention coherence varied. Most midwives articulated the LCG’s purpose following prior training, but conceptual clarity often exceeded procedural competence. Challenges were concentrated in contraction assessment and unfamiliar notation, indicating a need for ongoing on-the-job support and refresher training. These domains align in the mechanisms that drive positive implementation outcomes (acceptability) and clinical benefits, including early complication detection and respectful woman-centered care. Altogether, perceived effectiveness, burden and intervention coherence shaped the overall acceptability of the LCG in routine practice and may influence the extent to which its intended benefits are realized.

## 4 Discussion

This study explored the acceptability of the WHO Labor Care Guide among midwives working in healthcare facilities. Overall, midwives perceived the LCG as acceptable and effective tool that could support labor monitoring and maternal and neonatal outcomes, offering a more structured and detailed workflow approach compared to the traditional partograph that has been in use for over five decades. Participants highlighted that the LCG promotes vigilant observation, timely detection of complications, and informed clinical decision-making, thereby enhancing the quality of intrapartum care. However, they also acknowledged practical challenges, for instance, its detailed nature can increase documentation workload, particularly under staffing constraints and during multiple concurrent labors, and certain components such as contraction assessment and unfamiliar notation require on-the-job training and refresher sessions for optimal use. These findings underscore that the acceptability and successful implementation of the LCG are shaped not only by perceived clinical benefits but also by contextual factors, including adequate training, sufficient staffing levels, manageable workloads and supportive supervision.

In our study, midwives consistently described the LCG as a structured guide that enhances attentiveness, supports timely clinical decision-making particularly through systematic fetal heart rate tracking, standardized labor progress assessments, and earlier recognition of complications. Importantly, these data reflect perceived mechanisms through which the LCG may support intrapartum care such as prompting vigilance, improving continuity of documentation across shifts, and supporting timely escalation rather than objective evidence of improved clinical outcomes. These viewpoints align with the multi-country mixed-methods study conducted by Vogel and colleagues, where 136 maternity care practitioners across six countries reported the LCG as usable, feasible and acceptable, emphasizing its role in precise monitoring, critical thinking, and woman-centered care (9). Likewise, the international survey conducted to explore opinions of skilled health personnel from Africa, Asia, Europe and Latin America that informed LCG development using a self-administered questionnaire, found that most variables were perceived as clear, relevant, and complete, with reference thresholds considered useful for triggering reflection and action to improve decision-making and respectful care (11). Our findings are broadly consistent with implementation-focused studies reporting that providers often perceive the LCG as a useful tool for structured monitoring and decision support, while also noting increased documentation demands during early uptake. Qualitative and mixed-method evaluations from other low- and middle-income settings similarly highlight that perceived benefits (structured monitoring and improved clinical reasoning) coexist with concerns about time, staffing constraints, and the need for training reinforcement particularly for components requiring precise observation and unfamiliar documentation conventions (9, 11). Rather than using experimental trial outcomes to validate participants’ perceptions, we interpret our data as experiential evidence about acceptability and the conditions required for sustained use in routine practice. Our findings therefore contribute context-specific indication on how frontline midwives experience the LCG as a decision-support tool during early routine adoption.

Perceived burden emerged strongly in relation to the LCG’s expanded documentation requirements and the time needed to complete sections during busy shifts. Within the TFA, these descriptions represent the perceived effort required to engage with the intervention and therefore form a central component of acceptability. In other words, workload and personnel limitations functioned as contextual conditions that shaped the experienced burden and, consequently, the overall acceptability of routine LCG use. These findings suggest that efforts to improve acceptability should not focus only on the form itself, but also on implementation support that reduces documentation friction such as workflow redesign, harmonization with registers, and adequate staffing coverage. These findings echo prior research from Nigeria where workload pressure, insufficient time, inadequate staff and documentation complexity were cited as barriers to LCG uptake (29). Our analysis suggests that burden is not inherent to the tool but contingent on contextual factors such as staffing ratios and workflow integration in healthcare service delivery. In our case, already the government had introduced LCG as standard to be used in all public health facilities and therefore triggers for overcoming barriers in different work contexts were necessary. As an alternative to this, we located a successful case from the Ugandan WHO- LCG customization study where health facility providers reached a complementary conclusion from the opposite direction, i.e., by co-designing format and workflow, for example, adding locally relevant fields, re-ordering sections, and creating a back page for outcomes, end-users rated the adapted LCG as appealing, easy to use, and, notably, fast to complete after assessments highlighting how contextual adaptation of LCG workflow can convert barrier into advantage (30). In this aspect, we might say that in some instances, LCG acceptability might appear contingent rather than intrinsic to the tool with staffing ratios, documentation integration, supplies, and supervision modulating the effort required. This is consistent with WHO guidance that LCG implementation should be accompanied by proper orientation and training, supportive supervision, and an enabling environment at health facilities across rural and urban settings.

Participants in this study articulated LCG objectives more or less clearly, linking the guide to improved maternal and neonatal outcomes and woman-centered care. At the same time, study participants’ views indicated that coherence was uneven at the procedural level. While many midwives demonstrated conceptual understanding of “what the LCG is for,” some reported difficulties with “how to complete specific sections correctly,” particularly contraction assessment within 10-min intervals, head descent tracking, and interpretation of unfamiliar notation. This suggests that intervention coherence during early implementation may be dynamic strengthening with training reinforcement, mentorship, and repeated use but may remain fragile when staffing constraints limit time for careful assessment and documentation. A more cautious interpretation is therefore warranted as coherence was present in principle but not uniformly established across all components of the LCG. This pattern resonates with the international survey where a few WHO – LCG variables such as coping, urine and neonatal status, drew mixed views among study respondents despite overall support for design and thresholds (11). It also aligns with early qualitative work among Indonesian midwives from three rural hospitals that documented reluctance to move away from the familiar partograph and called for robust implementation strategies including frequent training and shared decision making among healthcare providers and families (10, 31).

Our findings extend the emerging global evidence on the LCG acceptability by illustrating how acceptability in early routine adoption is co-produced by TFA attributes and the implementation environment in public primary-care maternity settings (15, 29, 30,). Midwives’ accounts suggest that perceived effectiveness may be strengthened when the LCG functions as a structured cognitive aid that promotes vigilant monitoring and supports clinical decision making in improving intrapartum care (9, 12). However, these potential benefits may be undermined when documentation demands exceed available staffing and when knowledge or skill gaps limit correct completion of technically demanding components (31). This underscores that scale-up strategies need to address both skills (to strengthen coherence) and system constraints (to reduce burden), rather than assuming acceptability is inherent to the LCG itself (30).

Reflecting on rather than through our analysis with global evidence, three priorities emerge as keys to LCG acceptability among midwives. First, there is great emphasis that training, mentorship and supportive supervision while implementing a new WHO- LCG tool helps to build implementation coherence. This might include a short refresher training on contraction timing, symbol interpretation, and second-stage labor monitoring, coupled with bedside coaching and audit-and-feedback. Similar observations are frequently flagged by WHO and trials as core to a successful LCG strategy (32). Second, the need for LCG tool workflow and documentation integration to reduce burden among health service providers. In this aspect, adaptation of local contexts might be necessary as exemplified in the Ugandan study (using, for example co-design with LCG end-users) where WHO – LCG modifications such as field re-ordering, back-page outcomes and harmonization with registers were accommodated (30). Finally, health facilities need to be equipped with resources and supervision to convert perceived effectiveness into realized outcomes such as adequate staffing, reliable supplies, and supportive supervision all contextual influences underlined by both the multi-country evaluation and WHO implementation materials (9, 33).

Variation in participants’ educational level, seniority, and facility context appeared to shape how the LCG was understood and experienced during WHO-LCG implementation. Midwives with degree-level training often described the tool as intuitive, structured, and consistent with evidence-based intrapartum monitoring. In contrast, midwives with certificate or diploma training particularly those whose pre-service education preceded the LCG more frequently reported difficulties with unfamiliar notation, contraction assessment procedures, and the expanded scope of documentation, suggesting that educational preparation may influence intervention coherence and perceived burden. Differences by seniority were also evident. For instance, less experienced midwives tended to view the LCG as a supportive decision aid that increased confidence during complex or high-risk labor stages, whereas more experienced midwives sometimes compared it to the traditional partograph and described greater disruption to established routines or increased administrative work. Taken together, these patterns indicate that perceived acceptability is not solely a function of the tool itself but is co-produced by workforce profile such as training and experience and local implementation conditions such as staffing and workload (34).

This study provides analytic insights that may be transferable to comparable low-resource, rural–urban; high-low health facility delivery primary-healthcare facilities providing intrapartum care settings. Although our participants included midwives with diverse educational levels and years of experience, all participants were recruited from public dispensaries and health centers within a single municipal. Therefore, perspectives may reflect early-stage adoption dynamics and local workflow realities, and may differ in higher-level healthcare facilities, private or faith-based settings, or regions with different training intensity and resource availability. Importantly, the purposeful inclusion of participants with varying levels of education and seniority strengthened the credibility of the analysis by capturing a broader range of user experiences and perspectives. However, it also implies that WHO- LCG acceptability may vary contextually across workforce subgroups.

This study employed a qualitative exploratory case study design, which, while appropriate for understanding midwives’ perceptions and experiences of using LCG, inherently limits the representativeness of findings beyond the specific context. Our study was conducted almost a year after LCG initiation and training, meaning that participants’ experiences may reflect initial effects, i.e., concurrent acceptability rather than long-term implementation (35). The absence of triangulation with observational or quantitative data including routinely reported challenges restricts the ability to objectively measure the actual effect of LCG on maternal and neonatal outcomes, which could have strengthened the rationality of findings (16). Contextual factors such as staffing shortages and resource constraints, which were frequently regarded as barriers, may differ across regions and rural–urban settings, limiting transferability even within Tanzania. Furthermore, there is a lack of longitudinal follow-up, which could have provided insights into uptake, coverage and changes in acceptability over time. Despite these limitations, the study presents valuable insights on midwives LCG acceptability in healthcare facilities and highlights critical areas in clinical practice to improve maternal health care within the context of public health. Future research should consider mixed methods approaches combining qualitative insights with quantitative measures of maternal and neonatal outcomes, as well as longitudinal designs to assess the evolution of LCG acceptability and its integration into routine practice. Expanding the sample to include diverse geographic and facility types would enhance representativeness and inform context-specific implementation strategies.

## 5 Conclusion

In conclusion, findings from our study show that the LCG was widely perceived as an acceptable tool that could support labor monitoring, hence improve maternal and neonatal health outcomes through structured labor monitoring and timely decision-making. Midwives acknowledged its role in improving the quality of intrapartum care, preventing complications, and fostering a woman-centered approach. Despite these positive perceptions, several implementation challenges were identified. These include increased documentation workload, insufficient staffing, and inadequate on-the-job training and supportive supervision. Our findings are likely transferable to similar public primary-care maternity settings with comparable health facility contexts such as rural–urban; high-low health facility delivery; staffing and workload constraints. Broader generalization should be made cautiously. Policy efforts should prioritize on-the-job training, workflow adaptation, and regular supportive supervision. Future studies are warranted and should consider mixed-method approaches combining qualitative insights with quantitative measures of maternal and neonatal outcomes, routine data as well as longitudinal designs with to assess the progression of LCG acceptability and its integration into routine practice.

## Data Availability

The raw data supporting the conclusions of this article will be made available by the authors, without undue reservation.
