# Supplementary material for: Acceptability of the WHO labor care guide among midwives in Tanzania: an exploratory qualitative study
Source: Front Public Health. 2026 Mar 16;14:1739655. doi: 10.3389/fpubh.2026.1739655 (PMC13034876; doi:10.3389/fpubh.2026.1739655)
Supplement: Supplementary file 2 [file Supplementary_file_2.docx]

**Supplementary File S2 (EXTRACT of interview guide- English version)**

**Study:** Acceptability of the WHO - Labour Care Guide (LCG) among midwives in Tanzania

**Method:** In-depth interviews guided by the Theoretical Framework of Acceptability (TFA)

**NOTE: *Introductory parts have been removed and information contained in Part 1. Only the relevant part of the interview guide for this manuscript is shown.***

- Facility type: ☐ Dispensary ☐ Health center
- Approx. number of midwives on a typical shift: ____
- Average number of women giving birth per shift (approx.): ____

**A2. Professional background**

1. Can you tell me about your professional background and your current role in this facility?

*Probes: What are your main duties during labour and delivery?*

2. How long have you been working as a midwife (overall and in this facility)?

3. Do you know what Labour Care Guide is?, can you briefly describe it in your own words?

**Probes**: what is it used for?

- LCG content and structure?
- When did it start to be introduced?
- Difference between the LCG and traditional Partogram.

4. Have you received any training or orientation on the LCG?

**Probes***: When and by whom?*

- *Was it practical or mostly theoretical?*
- *Any refresher training?*

4. How long have you been using the LCG in routine care?

Probe: Do you use it for all women in labour, or only some? Why?

**SECTION B: Perceived effectiveness**

1. In your experience, how has using the LCG affected the way you monitor labor and make decisions?

**Probes:**

- *Which parts/sections of the LCG are most helpful for decision-making?*
- *Can you describe a situation where the LCG helped you notice a problem earlier?*
- *Has the LCG changed how you respond to slow progress, fetal distress, or other complications?*
- *How does the LCG affect communication and continuity of care across shifts?*

2. What aspects of the LCG do you find useful in monitoring labour? Please explain.

*Probes: fetal heart rate, contractions, cervical dilation, blood pressure, postpartum monitoring.*

3. Compared with the previous tool (partograph), what is better or worse about the LCG in supporting clinical care?

*Probes: What does the LCG add? What did the partograph do better?*

4. In what ways, if any, has the LCG influenced the quality of care provided during labour?

*Probes: timeliness, teamwork, completeness, woman-centred aspects (e.g., companionship, counselling).*

**SECTION C: Burden**

1. What effort or extra work, if any, does the LCG require compared with the partograph?

Probes- *as interview flows...*

- *Which sections take the most time to complete?*
- *How do staffing levels or multiple labouring women affect your ability to complete the LCG?*
- *What do you do when you prioritize urgent clinical care over documentation?*
- *Are there times you complete the LCG later? What are the risks/challenges of that?*

2. Do you feel that using the LCG has increased your workload? If yes, in what ways?

3. How does the time spent on LCG documentation affect the care you provide to women in labour?

**Probes***: delays, interruptions, perceived neglect by women, missed monitoring.*

4. What makes using the LCG more challenging in your daily work?

*Probes: staff shortage, high caseload, supplies/equipment, competing registers, shift handover.*

5. Are there challenges with how the LCG fits with facility protocols or reporting systems?

**Probes:** duplication with registers, requirement to fill both tools, filing/storage, supervision, audits.

**SECTION D: Intervention coherence**

1. What do you understand to be the main purpose of the LCG, and how is it supposed to work in practice?

**Probes**: *Which parts are clear or unclear to you?*

- *Are there symbols or steps that are confusing?*
- *How did training prepare you to use the LCG?*
- *What additional support would improve your confidence in using it?*

2. Do you feel able to follow the LCG steps in sequence (chronologically) during routine care?

**Probes:** *When is it difficult? What do you do in emergencies or when a woman arrives fully dilated?*

3. Are there any components you find difficult to implement in practice?

**Probes***: contraction assessment (10-minute interval and duration), fetal head descent, notation (> / <), second stage monitoring.*

4. Do you feel the LCG is consistent with labour care processes you are familiar with? Why or why not?

**Probes***: clinical fit, workflow fit, interaction with partograph routines.*

**SECTION E: Implementation of conditions and recommendations**

1. What conditions would make it easier to use the LCG as intended in your facility?

**Probes***: staffing, workflow changes, training/refreshers, supervision/mentorship, supplies, integration with registers.*

2. What recommendations would you make to improve LCG implementation in facilities like yours?

3. Is there anything else about your experience using the LCG that we have not discussed but you think is important?
